# 3-(2-Hy­droxy­phen­yl)-1-{(*E*)-[1-(pyrazin-2-yl)ethyl­idene]amino}­thio­urea monohydrate

**DOI:** 10.1107/S1600536811010038

**Published:** 2011-03-23

**Authors:** Erna Normaya, Yang Farina, Siti Nadiah Abd Halim, Edward R. T. Tiekink

**Affiliations:** a International Islamic University Malaysia (IIUM), PO Box 141, Kuantan, Malaysia; b School of Chemical Sciences and Food Technology, Faculty Science and Technology, Universiti Kebangsaan Malaysia, 43600 UKM Bangi, Selangor, Malaysia; c Department of Chemistry, University of Malaya, 50603 Kuala Lumpur, Malaysia

## Abstract

In the title compound, C_13_H_13_N_5_OS·H_2_O, the thio­urea mol­ecules closely resemble each other and are approximately planar; the dihedral angles formed between the terminal benzene rings are 7.88 (8) and 7.20 (8)°, respectively. The observed planarity correlates with the presence of bifurcated N—H⋯(O,N) hydrogen bonds. In the crystal, the mol­ecules are connected into supra­molecular double chains *via* a combination of N—H⋯S (linking the two independent mol­ecules), O—H⋯O and O—H⋯N (linking dimeric aggregates into a supra­molecular chain *via* hy­droxy–water, water–water and water–pyrazine inter­actions) and O—H⋯S hydrogen bonds (connecting two chains). The chains are further connected by C—H⋯N and C—H⋯S inter­actions.

## Related literature

For biological activity of thio­urea derivatives, see: Venkatachalam *et al.* (2004 ▶). For related structures, see: Gunasekaran *et al.* (2010 ▶); Dzulkifli *et al.* (2011 ▶). For additional geometric analysis, see: Spek (2009 ▶).
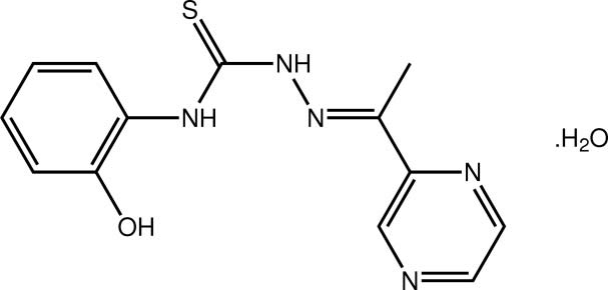


## Experimental

### 

#### Crystal data

C_13_H_13_N_5_OS·H_2_O
*M* *_r_* = 305.36
Triclinic, 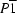

*a* = 7.9808 (5) Å
*b* = 11.7557 (8) Å
*c* = 16.4160 (11) Å
α = 99.638 (1)°
β = 94.128 (1)°
γ = 109.200 (1)°
*V* = 1420.54 (16) Å^3^
*Z* = 4
Mo *K*α radiation
μ = 0.24 mm^−1^
*T* = 100 K
0.18 × 0.14 × 0.11 mm

#### Data collection

Bruker SMART APEX diffractometer
Absorption correction: multi-scan (*SADABS*; Sheldrick, 1996 ▶) *T*_min_ = 0.656, *T*_max_ = 0.746
18244 measured reflections
6505 independent reflections
5267 reflections with *I* > 2σ(*I*)
*R*_int_ = 0.031

#### Refinement

*R*[*F* ^2^ > 2σ(*F* ^2^)] = 0.035
*wR*(*F* ^2^) = 0.110
*S* = 1.04
6505 reflections
411 parameters
12 restraints
H atoms treated by a mixture of independent and constrained refinement
Δρ_max_ = 0.39 e Å^−3^
Δρ_min_ = −0.30 e Å^−3^

### 

Data collection: *APEX2* (Bruker, 2008 ▶); cell refinement: *SAINT* (Bruker, 2008 ▶); data reduction: *SAINT*; program(s) used to solve structure: *SHELXS97* (Sheldrick, 2008 ▶); program(s) used to refine structure: *SHELXL97* (Sheldrick, 2008 ▶); molecular graphics: *ORTEP-3* (Farrugia, 1997 ▶), *DIAMOND* (Brandenburg, 2006 ▶) and *Qmol* (Gans & Shalloway, 2001 ▶); software used to prepare material for publication: *publCIF* (Westrip, 2010 ▶).

## Supplementary Material

Crystal structure: contains datablocks global, I. DOI: 10.1107/S1600536811010038/hg5012sup1.cif

Structure factors: contains datablocks I. DOI: 10.1107/S1600536811010038/hg5012Isup2.hkl

Additional supplementary materials:  crystallographic information; 3D view; checkCIF report

## Figures and Tables

**Table 1:** Hydrogen-bond geometry (Å, °)

*D*—H⋯*A*	*D*—H	H⋯*A*	*D*⋯*A*	*D*—H⋯*A*
N1—H1n⋯O1	0.88 (1)	2.11 (2)	2.5720 (15)	112 (1)
N1—H1n⋯N3	0.88 (1)	2.04 (2)	2.5435 (18)	116 (1)
N6—H6n⋯O2	0.87 (1)	2.11 (2)	2.5676 (15)	112 (1)
N6—H6n⋯N8	0.87 (1)	2.02 (2)	2.5358 (17)	117 (1)
O1—H1o⋯O1w	0.83 (1)	1.86 (1)	2.6820 (15)	170 (2)
O2—H2o⋯O2w^i^	0.83 (1)	1.83 (1)	2.6481 (16)	169 (2)
O1w—H1w⋯N9^ii^	0.84 (1)	1.96 (1)	2.7958 (17)	169 (2)
O1w—H2w⋯S2^iii^	0.83 (2)	2.82 (2)	3.4648 (13)	136 (2)
O2w—H3w⋯N4	0.84 (1)	2.02 (1)	2.8547 (17)	171 (2)
O2w—H4w⋯O1w	0.85 (2)	2.00 (2)	2.8357 (18)	169 (2)
N2—H2n⋯S2^iv^	0.87 (1)	2.67 (1)	3.4802 (12)	156 (1)
N7—H7n⋯S1^v^	0.87 (1)	2.58 (1)	3.4508 (12)	176 (2)
C16—H16⋯N5^vi^	0.95	2.58	3.517 (2)	172
C22—H22a⋯S1^v^	0.98	2.79	3.4454 (16)	125

Symmetry codes: (i) 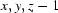; (ii) 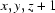; (iii) 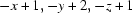; (iv) 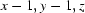; (v) 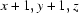; (vi) 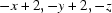.

**Fig. 1.**
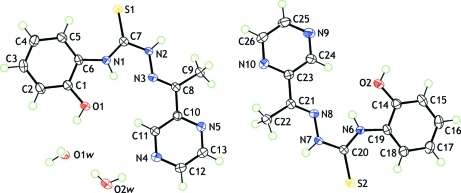
The molecular structures of the components defining the asymmetric unit of (I) showing the atom-labelling scheme and displacement ellipsoids at the 70% probability level.

**Fig. 2.**
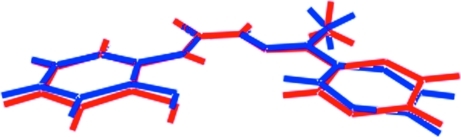
Overlay diagram showing the superimposition of the S1-containing molecule (red) with the S2-containing molecule (blue).

**Fig. 3.**
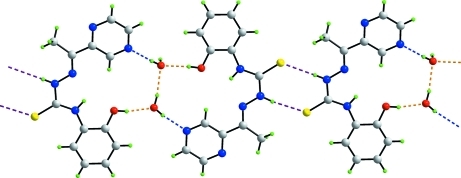
Supramolecular chain in (I) mediated by O—H···O (orange), O—H···N (blue) and N—H···S (purple) hydrogen bonding shown as dashed lines.

**Fig. 4.**
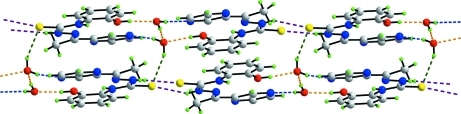
Supramolecular double chain in (I) whereby the chain in Fig. 3 is connected via O—H···S hydrogen bonds shown as green dashed lines.

**Fig. 5.**
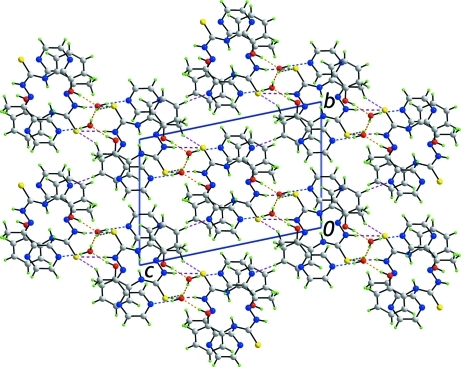
View in projection down the a axis of the crystal packing for (I). The C—H···N and C—H···S contacts are shown as pink dashed lines.

## supplementary crystallographic information

### Comment

Thiourea derivatives have biological potential (Venkatachalam *et al.*, 2004) and attract continuing structural studies (Gunasekaran *et al.*, 2010; Dzulkifli *et al.*, 2011).

The title compound (I), Fig. 1, features two independent thiourea derivatives and two water molecules of crystallization in the asymmetric unit. As seen from the overlay diagram, Fig. 2, there are small differences between the independent thiourea molecules. These relate to the relative orientations of the terminal benzene rings; the r.m.s. deviation of bond distances = 0.0032 Å (Spek, 2009). The similarity between the molecules is seen in the dihedral angle formed between the rings = 7.88 (8) ° for the S1-containing molecule and 7.20 (8) ° for the other. The planarity of each molecule is readily explained in terms of bifurcated intramolecular N—H···O,*N* hydrogen bonds, Table 1.

The most prominent feature of the crystal packing is the formation of supramolecular double chains along [111]. The two molecules comprising the asymmetric unit are connected *via* an eight-membered {···HNC═S}_2_ synthon and the resulting dimeric aggregates are connected by two molecules of water *via* a sequence of O—H···O hydrogen bonds. Thus, the hydroxyl group of one molecule is connected to a water molecule which hydrogen bonds to the second water molecule which in turn links the second hydroxyl group. Hydrogen bonds between the water molecules and pyrazine-N atoms close two fused 16-membered {···HOH···NC_3_N_2_CNC_2_OH···O} synthons. This arrangement is stabilized by the intramolecular interactions outlined above. The result is a supramolecular chain, Fig. 3. Pairs of chains are linked *via* water-O—H···S hydrogen bonds leading to a double chain, Fig. 4. The chains are consolidated in the crystal packing by C—H···N and C—H···S interactions, Table 1 and Fig. 5.

### Experimental

The reaction of 2-acetylpyrazine with methyl hydrazinecarbodithioate (II) formed (*E*)-methyl-2-(1-(pyrazin-2-yl)ethylidene)hydrazinecarbodithioate (III). The condensation reaction of (III) with 2-phenolamine produced the title compound, (I), *i.e*. (*E*)-*N*-(2-hydroxyphenyl)-2-(1-(pyrazin-2- yl)ethylidene)hydrazinecarbothioamide (yield:73.3%, *M*.pt. 469—471 K).

Slow recrystallization of its butanol solution afforded yellow crystals of (I). Elemental anal. (calc.): C, 51.80 (51.13); H, 4.91 (4.95), N, 24.10 (22.94) %. FT—IR (ν_max_; cm^-1^): (O—H) 3459, (N—H) 3209, (C\b C) 3016, (CH_3_) 2925, (C═N) 1609, (C≐C aromatic) 1555, (C—N) 1366, (pyrazyl) 1162, (N —N) 1121 and (C═S) 1023.

### Refinement

Carbon-bound H-atoms were placed in calculated positions (C—H 0.95 to 0.98 Å) and were included in the refinement in the riding model approximation, with *U*_iso_(H) set to 1.2 to 1.5*U*_equiv_(C). The water-H and amine-H atoms were refined with the distance restraints O—H = 0.84±0.01 Å and N–H = 0.88±0.01 Å, and with *U*_iso_(H) = *yU*_equiv_(parent atom); *y* = 1.5 for O, and 1.2 for N. In addition, each pair of water-H atoms were constrained to be separated by 1.39±0.02 Å. Owing to poor agreement, the reflections (37 13) and (3 6 14) were omitted from the final refinement.

### Crystal data

**Table:**

C_13_H_13_N_5_OS·H_2_O	*Z* = 4
*M**_r_* = 305.36	*F*(000) = 640
Triclinic, *P*1	*D*_x_ = 1.428 Mg m^−^^3^
Hall symbol: -P 1	Mo *K*α radiation, λ = 0.71073 Å
*a* = 7.9808 (5) Å	Cell parameters from 6288 reflections
*b* = 11.7557 (8) Å	θ = 2.5–30.6°
*c* = 16.4160 (11) Å	µ = 0.24 mm^−^^1^
α = 99.638 (1)°	*T* = 100 K
β = 94.128 (1)°	Block, yellow
γ = 109.200 (1)°	0.18 × 0.14 × 0.11 mm
*V* = 1420.54 (16) Å^3^	

### Data collection

**Table:**

Bruker SMART APEX diffractometer	6505 independent reflections
Radiation source: fine-focus sealed tube	5267 reflections with *I* > 2σ(*I*)
graphite	*R*_int_ = 0.031
ω scans	θ_max_ = 27.5°, θ_min_ = 1.3°
Absorption correction: multi-scan (*SADABS*; Sheldrick, 1996)	*h* = −10→10
*T*_min_ = 0.656, *T*_max_ = 0.746	*k* = −15→15
18244 measured reflections	*l* = −21→21

### Refinement

**Table:**

Refinement on *F*^2^	Primary atom site location: structure-invariant direct methods
Least-squares matrix: full	Secondary atom site location: difference Fourier map
*R*[*F*^2^ > 2σ(*F*^2^)] = 0.035	Hydrogen site location: inferred from neighbouring sites
*wR*(*F*^2^) = 0.110	H atoms treated by a mixture of independent and constrained refinement
*S* = 1.04	*w* = 1/[σ^2^(*F*_o_^2^) + (0.0586*P*)^2^ + 0.4197*P*] where *P* = (*F*_o_^2^ + 2*F*_c_^2^)/3
6505 reflections	(Δ/σ)_max_ = 0.001
411 parameters	Δρ_max_ = 0.39 e Å^−^^3^
12 restraints	Δρ_min_ = −0.30 e Å^−^^3^

### Special details

**Table:**

Geometry. All s.u.'s (except the s.u. in the dihedral angle between two l.s. planes) are estimated using the full covariance matrix. The cell s.u.'s are taken into account individually in the estimation of s.u.'s in distances, angles and torsion angles; correlations between s.u.'s in cell parameters are only used when they are defined by crystal symmetry. An approximate (isotropic) treatment of cell s.u.'s is used for estimating s.u.'s involving l.s. planes.
Refinement. Refinement of *F*^2^ against ALL reflections. The weighted *R*-factor *wR* and goodness of fit *S* are based on *F*^2^, conventional *R*-factors *R* are based on *F*, with *F* set to zero for negative *F*^2^. The threshold expression of *F*^2^ > 2σ(*F*^2^) is used only for calculating *R*-factors(gt) *etc*. and is not relevant to the choice of reflections for refinement. *R*-factors based on *F*^2^ are statistically about twice as large as those based on *F*, and *R*- factors based on ALL data will be even larger.

### Fractional atomic coordinates and isotropic or equivalent isotropic displacement parameters (Å^2^)

**Table:**

	*x*	*y*	*z*	*U*_iso_*/*U*_eq_	
S1	−0.09788 (5)	0.17115 (3)	0.34884 (2)	0.02054 (11)	
O1	0.19179 (14)	0.50524 (9)	0.62276 (6)	0.0191 (2)	
H1O	0.223 (2)	0.5485 (15)	0.6707 (7)	0.029*	
N1	0.06763 (16)	0.35848 (11)	0.48035 (7)	0.0160 (3)	
H1N	0.1553 (18)	0.4298 (10)	0.4942 (10)	0.019*	
N2	0.17706 (16)	0.37485 (11)	0.35765 (7)	0.0155 (3)	
H2N	0.176 (2)	0.3458 (15)	0.3051 (6)	0.019*	
N3	0.30025 (16)	0.48289 (11)	0.40124 (7)	0.0151 (2)	
N4	0.63006 (17)	0.80104 (11)	0.55376 (8)	0.0187 (3)	
N5	0.65300 (16)	0.74828 (11)	0.38282 (8)	0.0173 (3)	
C1	0.04480 (19)	0.40506 (13)	0.62335 (9)	0.0157 (3)	
C2	−0.0340 (2)	0.38248 (14)	0.69423 (9)	0.0193 (3)	
H2	0.0134	0.4390	0.7459	0.023*	
C3	−0.1821 (2)	0.27734 (14)	0.68991 (9)	0.0201 (3)	
H3	−0.2360	0.2619	0.7386	0.024*	
C4	−0.2515 (2)	0.19499 (14)	0.61453 (9)	0.0194 (3)	
H4	−0.3522	0.1228	0.6119	0.023*	
C5	−0.17480 (19)	0.21715 (13)	0.54249 (9)	0.0175 (3)	
H5	−0.2234	0.1606	0.4910	0.021*	
C6	−0.02660 (19)	0.32252 (13)	0.54647 (8)	0.0148 (3)	
C7	0.05216 (19)	0.30635 (13)	0.39999 (9)	0.0153 (3)	
C8	0.42014 (19)	0.55019 (13)	0.36458 (8)	0.0147 (3)	
C9	0.4445 (2)	0.52302 (14)	0.27451 (8)	0.0187 (3)	
H9A	0.4149	0.4342	0.2557	0.028*	
H9B	0.5692	0.5659	0.2680	0.028*	
H9C	0.3653	0.5512	0.2410	0.028*	
C10	0.53888 (18)	0.66744 (13)	0.41919 (9)	0.0146 (3)	
C11	0.52873 (19)	0.69459 (13)	0.50503 (9)	0.0167 (3)	
H11	0.4468	0.6349	0.5290	0.020*	
C12	0.7435 (2)	0.88265 (14)	0.51665 (9)	0.0201 (3)	
H12	0.8179	0.9601	0.5491	0.024*	
C13	0.7538 (2)	0.85604 (14)	0.43227 (10)	0.0210 (3)	
H13	0.8353	0.9162	0.4084	0.025*	
S2	1.07562 (5)	1.30651 (3)	0.14135 (2)	0.01929 (10)	
O2	0.78422 (15)	0.96845 (10)	−0.13104 (6)	0.0210 (2)	
H2O	0.746 (2)	0.9237 (16)	−0.1782 (7)	0.032*	
N6	0.91194 (16)	1.11695 (11)	0.01014 (7)	0.0157 (2)	
H6N	0.8277 (18)	1.0449 (10)	−0.0021 (10)	0.019*	
N7	0.80922 (16)	1.09918 (11)	0.13407 (7)	0.0157 (3)	
H7N	0.829 (2)	1.1201 (15)	0.1885 (6)	0.019*	
N8	0.68925 (16)	0.98922 (10)	0.09106 (7)	0.0150 (2)	
N9	0.35318 (18)	0.67259 (12)	−0.06046 (8)	0.0230 (3)	
N10	0.34629 (17)	0.72072 (12)	0.11192 (8)	0.0194 (3)	
C14	0.92330 (19)	1.07099 (13)	−0.13441 (9)	0.0164 (3)	
C15	0.9944 (2)	1.09597 (14)	−0.20668 (9)	0.0201 (3)	
H15	0.9453	1.0394	−0.2581	0.024*	
C16	1.1376 (2)	1.20380 (14)	−0.20400 (10)	0.0212 (3)	
H16	1.1856	1.2213	−0.2536	0.025*	
C17	1.2101 (2)	1.28569 (14)	−0.12866 (10)	0.0212 (3)	
H17	1.3081	1.3591	−0.1271	0.025*	
C18	1.1413 (2)	1.26184 (14)	−0.05531 (9)	0.0185 (3)	
H18	1.1926	1.3182	−0.0039	0.022*	
C19	0.99712 (19)	1.15496 (13)	−0.05791 (8)	0.0154 (3)	
C20	0.93076 (18)	1.16914 (13)	0.09055 (8)	0.0143 (3)	
C21	0.57203 (19)	0.92136 (13)	0.12853 (9)	0.0157 (3)	
C22	0.5460 (2)	0.94878 (14)	0.21817 (9)	0.0215 (3)	
H22A	0.5889	1.0380	0.2386	0.032*	
H22B	0.6137	0.9122	0.2512	0.032*	
H22C	0.4185	0.9141	0.2233	0.032*	
C23	0.45390 (19)	0.80368 (13)	0.07433 (9)	0.0158 (3)	
C24	0.4564 (2)	0.77884 (14)	−0.01203 (9)	0.0200 (3)	
H24	0.5342	0.8397	−0.0367	0.024*	
C25	0.2466 (2)	0.58978 (14)	−0.02229 (10)	0.0238 (3)	
H25	0.1707	0.5126	−0.0547	0.029*	
C26	0.2442 (2)	0.61369 (14)	0.06273 (10)	0.0227 (3)	
H26	0.1674	0.5520	0.0872	0.027*	
O1W	0.29296 (15)	0.66684 (11)	0.76876 (7)	0.0240 (3)	
H1W	0.320 (3)	0.6634 (19)	0.8187 (7)	0.036*	
H2W	0.209 (2)	0.6932 (18)	0.7662 (11)	0.036*	
O2W	0.62751 (16)	0.80846 (11)	0.72836 (7)	0.0264 (3)	
H4W	0.5218 (15)	0.7672 (16)	0.7342 (11)	0.040*	
H3W	0.634 (3)	0.8147 (19)	0.6783 (7)	0.040*	

### Atomic displacement parameters (Å^2^)

**Table:**

	*U*^11^	*U*^22^	*U*^33^	*U*^12^	*U*^13^	*U*^23^
S1	0.0241 (2)	0.01728 (19)	0.01090 (18)	−0.00349 (15)	0.00142 (14)	−0.00018 (13)
O1	0.0207 (5)	0.0182 (5)	0.0111 (5)	−0.0003 (4)	0.0027 (4)	−0.0024 (4)
N1	0.0165 (6)	0.0149 (6)	0.0117 (6)	−0.0001 (5)	0.0033 (5)	0.0003 (5)
N2	0.0180 (6)	0.0140 (6)	0.0091 (5)	−0.0001 (5)	0.0020 (5)	−0.0006 (5)
N3	0.0156 (6)	0.0141 (6)	0.0125 (6)	0.0027 (5)	0.0002 (5)	0.0000 (5)
N4	0.0196 (6)	0.0176 (6)	0.0157 (6)	0.0040 (5)	−0.0003 (5)	0.0009 (5)
N5	0.0162 (6)	0.0180 (6)	0.0154 (6)	0.0026 (5)	0.0029 (5)	0.0038 (5)
C1	0.0171 (7)	0.0164 (7)	0.0136 (7)	0.0060 (5)	0.0022 (5)	0.0025 (5)
C2	0.0243 (8)	0.0221 (7)	0.0125 (7)	0.0089 (6)	0.0043 (6)	0.0033 (6)
C3	0.0241 (8)	0.0241 (8)	0.0159 (7)	0.0106 (6)	0.0090 (6)	0.0075 (6)
C4	0.0191 (7)	0.0189 (7)	0.0211 (7)	0.0054 (6)	0.0078 (6)	0.0064 (6)
C5	0.0181 (7)	0.0181 (7)	0.0144 (7)	0.0050 (6)	0.0022 (5)	0.0006 (6)
C6	0.0175 (7)	0.0168 (7)	0.0113 (6)	0.0070 (6)	0.0043 (5)	0.0033 (5)
C7	0.0161 (7)	0.0162 (7)	0.0127 (7)	0.0047 (5)	0.0012 (5)	0.0025 (5)
C8	0.0165 (7)	0.0154 (7)	0.0113 (6)	0.0049 (5)	0.0013 (5)	0.0019 (5)
C9	0.0213 (7)	0.0202 (7)	0.0112 (7)	0.0034 (6)	0.0034 (6)	0.0017 (6)
C10	0.0138 (7)	0.0158 (7)	0.0140 (7)	0.0048 (5)	0.0017 (5)	0.0033 (5)
C11	0.0189 (7)	0.0157 (7)	0.0133 (7)	0.0035 (6)	0.0007 (5)	0.0027 (5)
C12	0.0202 (7)	0.0152 (7)	0.0200 (7)	0.0015 (6)	0.0000 (6)	0.0010 (6)
C13	0.0192 (7)	0.0184 (7)	0.0211 (8)	0.0008 (6)	0.0017 (6)	0.0046 (6)
S2	0.0241 (2)	0.01506 (18)	0.01128 (18)	−0.00166 (14)	0.00061 (14)	0.00055 (13)
O2	0.0234 (6)	0.0209 (5)	0.0113 (5)	0.0005 (4)	0.0013 (4)	−0.0016 (4)
N6	0.0174 (6)	0.0131 (6)	0.0116 (6)	−0.0005 (5)	0.0031 (5)	0.0001 (5)
N7	0.0198 (6)	0.0135 (6)	0.0093 (5)	0.0010 (5)	0.0018 (5)	−0.0003 (5)
N8	0.0170 (6)	0.0118 (6)	0.0135 (6)	0.0026 (5)	0.0008 (5)	0.0004 (4)
N9	0.0229 (7)	0.0214 (7)	0.0180 (6)	0.0018 (5)	0.0023 (5)	−0.0022 (5)
N10	0.0179 (6)	0.0185 (6)	0.0187 (6)	0.0013 (5)	0.0022 (5)	0.0057 (5)
C14	0.0183 (7)	0.0169 (7)	0.0150 (7)	0.0073 (6)	0.0030 (5)	0.0029 (6)
C15	0.0251 (8)	0.0255 (8)	0.0117 (7)	0.0116 (6)	0.0033 (6)	0.0032 (6)
C16	0.0264 (8)	0.0259 (8)	0.0181 (7)	0.0138 (7)	0.0106 (6)	0.0097 (6)
C17	0.0223 (8)	0.0212 (7)	0.0225 (8)	0.0076 (6)	0.0088 (6)	0.0079 (6)
C18	0.0196 (7)	0.0189 (7)	0.0167 (7)	0.0057 (6)	0.0052 (6)	0.0034 (6)
C19	0.0187 (7)	0.0182 (7)	0.0110 (6)	0.0079 (6)	0.0037 (5)	0.0035 (5)
C20	0.0158 (7)	0.0146 (6)	0.0114 (6)	0.0042 (5)	0.0015 (5)	0.0021 (5)
C21	0.0173 (7)	0.0167 (7)	0.0127 (7)	0.0054 (6)	0.0019 (5)	0.0029 (5)
C22	0.0231 (8)	0.0217 (8)	0.0136 (7)	0.0011 (6)	0.0044 (6)	0.0003 (6)
C23	0.0150 (7)	0.0160 (7)	0.0153 (7)	0.0040 (5)	0.0018 (5)	0.0027 (5)
C24	0.0202 (7)	0.0194 (7)	0.0154 (7)	0.0015 (6)	0.0021 (6)	0.0006 (6)
C25	0.0215 (8)	0.0166 (7)	0.0264 (8)	0.0008 (6)	0.0015 (6)	−0.0016 (6)
C26	0.0194 (7)	0.0188 (7)	0.0248 (8)	−0.0001 (6)	0.0017 (6)	0.0055 (6)
O1W	0.0261 (6)	0.0278 (6)	0.0161 (5)	0.0115 (5)	−0.0008 (5)	−0.0039 (5)
O2W	0.0304 (6)	0.0284 (6)	0.0128 (5)	0.0032 (5)	0.0014 (5)	−0.0014 (5)

### Geometric parameters (Å, °)

**Table:**

S1—C7	1.6797 (14)	O2—H2O	0.832 (9)
O1—C1	1.3638 (17)	N6—C20	1.3347 (17)
O1—H1O	0.834 (9)	N6—C19	1.4094 (18)
N1—C7	1.3370 (18)	N6—H6N	0.871 (9)
N1—C6	1.4087 (18)	N7—N8	1.3671 (16)
N1—H1N	0.878 (9)	N7—C20	1.3782 (18)
N2—N3	1.3656 (16)	N7—H7N	0.874 (9)
N2—C7	1.3727 (18)	N8—C21	1.2830 (18)
N2—H2N	0.872 (9)	N9—C24	1.3294 (19)
N3—C8	1.2837 (18)	N9—C25	1.342 (2)
N4—C11	1.3289 (18)	N10—C26	1.3379 (19)
N4—C12	1.3471 (19)	N10—C23	1.3403 (18)
N5—C10	1.3372 (18)	C14—C15	1.386 (2)
N5—C13	1.3425 (19)	C14—C19	1.4122 (19)
C1—C2	1.385 (2)	C15—C16	1.392 (2)
C1—C6	1.4078 (19)	C15—H15	0.9500
C2—C3	1.389 (2)	C16—C17	1.387 (2)
C2—H2	0.9500	C16—H16	0.9500
C3—C4	1.387 (2)	C17—C18	1.392 (2)
C3—H3	0.9500	C17—H17	0.9500
C4—C5	1.394 (2)	C18—C19	1.389 (2)
C4—H4	0.9500	C18—H18	0.9500
C5—C6	1.391 (2)	C21—C23	1.4844 (19)
C5—H5	0.9500	C21—C22	1.4972 (19)
C8—C10	1.4854 (19)	C22—H22A	0.9800
C8—C9	1.5000 (19)	C22—H22B	0.9800
C9—H9A	0.9800	C22—H22C	0.9800
C9—H9B	0.9800	C23—C24	1.402 (2)
C9—H9C	0.9800	C24—H24	0.9500
C10—C11	1.4061 (19)	C25—C26	1.380 (2)
C11—H11	0.9500	C25—H25	0.9500
C12—C13	1.383 (2)	C26—H26	0.9500
C12—H12	0.9500	O1W—H1W	0.845 (9)
C13—H13	0.9500	O1W—H2W	0.829 (9)
S2—C20	1.6799 (14)	O2W—H4W	0.845 (9)
O2—C14	1.3562 (18)	O2W—H3W	0.842 (9)
			
C1—O1—H1O	109.1 (13)	C20—N6—C19	133.48 (12)
C7—N1—C6	133.34 (12)	C20—N6—H6N	111.9 (11)
C7—N1—H1N	112.4 (11)	C19—N6—H6N	114.6 (11)
C6—N1—H1N	114.2 (11)	N8—N7—C20	117.62 (11)
N3—N2—C7	117.93 (11)	N8—N7—H7N	123.7 (11)
N3—N2—H2N	123.7 (11)	C20—N7—H7N	117.2 (11)
C7—N2—H2N	118.4 (11)	C21—N8—N7	120.02 (12)
C8—N3—N2	120.36 (12)	C24—N9—C25	116.48 (13)
C11—N4—C12	116.55 (13)	C26—N10—C23	116.37 (13)
C10—N5—C13	116.46 (13)	O2—C14—C15	124.21 (13)
O1—C1—C2	123.55 (13)	O2—C14—C19	115.96 (12)
O1—C1—C6	116.29 (12)	C15—C14—C19	119.83 (14)
C2—C1—C6	120.16 (13)	C14—C15—C16	120.11 (14)
C1—C2—C3	120.07 (14)	C14—C15—H15	119.9
C1—C2—H2	120.0	C16—C15—H15	119.9
C3—C2—H2	120.0	C17—C16—C15	119.82 (14)
C4—C3—C2	119.96 (14)	C17—C16—H16	120.1
C4—C3—H3	120.0	C15—C16—H16	120.1
C2—C3—H3	120.0	C16—C17—C18	120.92 (15)
C3—C4—C5	120.61 (14)	C16—C17—H17	119.5
C3—C4—H4	119.7	C18—C17—H17	119.5
C5—C4—H4	119.7	C19—C18—C17	119.41 (14)
C6—C5—C4	119.63 (13)	C19—C18—H18	120.3
C6—C5—H5	120.2	C17—C18—H18	120.3
C4—C5—H5	120.2	C18—C19—N6	126.65 (13)
C5—C6—C1	119.56 (13)	C18—C19—C14	119.91 (13)
C5—C6—N1	127.05 (13)	N6—C19—C14	113.44 (12)
C1—C6—N1	113.39 (12)	N6—C20—N7	113.11 (12)
N1—C7—N2	113.40 (12)	N6—C20—S2	128.34 (11)
N1—C7—S1	127.44 (11)	N7—C20—S2	118.52 (10)
N2—C7—S1	119.15 (10)	N8—C21—C23	113.87 (12)
N3—C8—C10	114.04 (12)	N8—C21—C22	127.09 (13)
N3—C8—C9	126.55 (13)	C23—C21—C22	119.03 (12)
C10—C8—C9	119.37 (12)	C21—C22—H22A	109.5
C8—C9—H9A	109.5	C21—C22—H22B	109.5
C8—C9—H9B	109.5	H22A—C22—H22B	109.5
H9A—C9—H9B	109.5	C21—C22—H22C	109.5
C8—C9—H9C	109.5	H22A—C22—H22C	109.5
H9A—C9—H9C	109.5	H22B—C22—H22C	109.5
H9B—C9—H9C	109.5	N10—C23—C24	121.20 (13)
N5—C10—C11	121.07 (13)	N10—C23—C21	116.94 (12)
N5—C10—C8	117.13 (12)	C24—C23—C21	121.86 (13)
C11—C10—C8	121.77 (13)	N9—C24—C23	121.95 (14)
N4—C11—C10	122.14 (13)	N9—C24—H24	119.0
N4—C11—H11	118.9	C23—C24—H24	119.0
C10—C11—H11	118.9	N9—C25—C26	121.75 (14)
N4—C12—C13	121.38 (14)	N9—C25—H25	119.1
N4—C12—H12	119.3	C26—C25—H25	119.1
C13—C12—H12	119.3	N10—C26—C25	122.24 (14)
N5—C13—C12	122.38 (14)	N10—C26—H26	118.9
N5—C13—H13	118.8	C25—C26—H26	118.9
C12—C13—H13	118.8	H1W—O1W—H2W	109.0 (16)
C14—O2—H2O	111.0 (14)	H4W—O2W—H3W	110.5 (16)
			
C7—N2—N3—C8	179.84 (13)	C20—N7—N8—C21	−179.19 (13)
O1—C1—C2—C3	−179.12 (13)	O2—C14—C15—C16	−179.93 (14)
C6—C1—C2—C3	0.9 (2)	C19—C14—C15—C16	−0.3 (2)
C1—C2—C3—C4	−0.1 (2)	C14—C15—C16—C17	0.6 (2)
C2—C3—C4—C5	−0.6 (2)	C15—C16—C17—C18	−0.1 (2)
C3—C4—C5—C6	0.4 (2)	C16—C17—C18—C19	−0.6 (2)
C4—C5—C6—C1	0.4 (2)	C17—C18—C19—N6	179.92 (14)
C4—C5—C6—N1	179.51 (14)	C17—C18—C19—C14	0.8 (2)
O1—C1—C6—C5	178.95 (13)	C20—N6—C19—C18	5.0 (3)
C2—C1—C6—C5	−1.1 (2)	C20—N6—C19—C14	−175.84 (15)
O1—C1—C6—N1	−0.25 (18)	O2—C14—C19—C18	179.25 (13)
C2—C1—C6—N1	179.70 (13)	C15—C14—C19—C18	−0.4 (2)
C7—N1—C6—C5	−2.7 (3)	O2—C14—C19—N6	0.05 (18)
C7—N1—C6—C1	176.41 (15)	C15—C14—C19—N6	−179.61 (13)
C6—N1—C7—N2	−179.14 (14)	C19—N6—C20—N7	176.62 (14)
C6—N1—C7—S1	0.5 (2)	C19—N6—C20—S2	−1.4 (2)
N3—N2—C7—N1	1.30 (19)	N8—N7—C20—N6	0.04 (18)
N3—N2—C7—S1	−178.38 (10)	N8—N7—C20—S2	178.23 (10)
N2—N3—C8—C10	177.27 (12)	N7—N8—C21—C23	−178.68 (12)
N2—N3—C8—C9	−0.6 (2)	N7—N8—C21—C22	0.9 (2)
C13—N5—C10—C11	−0.9 (2)	C26—N10—C23—C24	0.7 (2)
C13—N5—C10—C8	177.13 (13)	C26—N10—C23—C21	−178.17 (13)
N3—C8—C10—N5	−172.44 (13)	N8—C21—C23—N10	170.53 (13)
C9—C8—C10—N5	5.6 (2)	C22—C21—C23—N10	−9.1 (2)
N3—C8—C10—C11	5.6 (2)	N8—C21—C23—C24	−8.3 (2)
C9—C8—C10—C11	−176.37 (13)	C22—C21—C23—C24	172.12 (14)
C12—N4—C11—C10	0.2 (2)	C25—N9—C24—C23	−0.3 (2)
N5—C10—C11—N4	0.4 (2)	N10—C23—C24—N9	−0.1 (2)
C8—C10—C11—N4	−177.57 (13)	C21—C23—C24—N9	178.71 (14)
C11—N4—C12—C13	−0.3 (2)	C24—N9—C25—C26	0.0 (2)
C10—N5—C13—C12	0.9 (2)	C23—N10—C26—C25	−0.9 (2)
N4—C12—C13—N5	−0.2 (2)	N9—C25—C26—N10	0.6 (3)

### Hydrogen-bond geometry (Å, °)

**Table:**

*D*—H···*A*	*D*—H	H···*A*	*D*···*A*	*D*—H···*A*
N1—H1n···O1	0.878 (13)	2.107 (16)	2.5720 (15)	112.3 (12)
N1—H1n···N3	0.878 (13)	2.038 (15)	2.5435 (18)	115.5 (13)
N6—H6n···O2	0.871 (12)	2.112 (16)	2.5676 (15)	111.9 (12)
N6—H6n···N8	0.871 (12)	2.023 (15)	2.5358 (17)	116.7 (13)
O1—H1o···O1w	0.833 (12)	1.857 (13)	2.6820 (15)	170.3 (16)
O2—H2o···O2w^i^	0.833 (13)	1.826 (14)	2.6481 (16)	168.9 (17)
O1w—H1w···N9^ii^	0.844 (13)	1.962 (12)	2.7958 (17)	169 (2)
O1w—H2w···S2^iii^	0.827 (18)	2.822 (18)	3.4648 (13)	136.0 (16)
O2w—H3w···N4	0.841 (13)	2.021 (12)	2.8547 (17)	171 (2)
O2w—H4w···O1w	0.845 (15)	2.001 (16)	2.8357 (18)	169.1 (16)
N2—H2n···S2^iv^	0.871 (10)	2.667 (10)	3.4802 (12)	155.9 (14)
N7—H7n···S1^v^	0.874 (10)	2.579 (10)	3.4508 (12)	175.6 (15)
C16—H16···N5^vi^	0.95	2.58	3.517 (2)	172
C22—H22a···S1^v^	0.98	2.79	3.4454 (16)	125

Symmetry codes: (i) *x*, *y*, *z*−1; (ii) *x*, *y*, *z*+1; (iii) −*x*+1, −*y*+2, −*z*+1; (iv) *x*−1, *y*−1, *z*; (v) *x*+1, *y*+1, *z*; (vi) −*x*+2, −*y*+2, −*z*.
